# Thymoquinone ameliorates pressure overload‐induced cardiac hypertrophy by activating the AMPK signalling pathway

**DOI:** 10.1111/jcmm.17138

**Published:** 2021-12-24

**Authors:** Heng Chen, Chengui Zhuo, Aohan Zu, Shuai Yuan, Han Zhang, Jianqiang Zhao, Liangrong Zheng

**Affiliations:** ^1^ Department of Cardiology and Atrial Fibrillation Center The First Affiliated Hospital College of Medicine Zhejiang University Hangzhou China; ^2^ Department of Cardiology Taizhou Central Hospital (Taizhou University Hospital) Taizhou Zhejiang China; ^3^ Echocardiography and Vascular Ultrasound Center The First Affiliated Hospital College of Medicine Zhejiang University Hangzhou China; ^4^ Department of Cardiology The Fourth Affiliated Hospital College of Medicine Zhejiang University Yiwu China

**Keywords:** AMPK, cardiac hypertrophy, fibrosis, heart failure, oxidative stress, thymoquinone

## Abstract

Prolonged pathological myocardial hypertrophy leads to end‐stage heart failure. Thymoquinone (TQ), a bioactive component extracted from *Nigella sativa* seeds, is extensively used in ethnomedicine to treat a broad spectrum of disorders. However, it remains unclear whether TQ protects the heart from pathological hypertrophy. This study was conducted to examine the potential utility of TQ for treatment of pathological cardiac hypertrophy and if so, to elucidate the underlying mechanisms. Male C57BL/6J mice underwent either transverse aortic constriction (TAC) or sham operation, followed by TQ treatment for six consecutive weeks. In vitro experiments consisted of neonatal rat cardiomyocytes (NRCMs) that were exposed to phenylephrine (PE) stimulation to induce cardiomyocyte hypertrophy. In this study, we observed that systemic administration of TQ preserved cardiac contractile function, and alleviated cardiac hypertrophy, fibrosis and oxidative stress in TAC‐challenged mice. The in vitro experiments showed that TQ treatment attenuated the PE‐induced hypertrophic response in NRCMs. Mechanistical experiments showed that supplementation of TQ induced reactivation of the AMP‐activated protein kinase (AMPK) with concomitant inhibition of ERK 1/2, p38 and JNK1/2 MAPK cascades. Furthermore, we demonstrated that compound C, an AMPK inhibitor, abolished the protective effects of TQ in in vivo and in vitro experiments. Altogether, our study disclosed that TQ provides protection against myocardial hypertrophy in an AMPK‐dependent manner and identified it as a promising agent for the treatment of myocardial hypertrophy.

## 1 INTRODUCTION

Cardiac hypertrophy is a compensatory response that mediates cardiac enlargement. Parallel addition of sarcomere units helps to increase cardiac contractility, at least initially.^1^ Hypertension, diabetes, myocardial infarction or other pathological conditions can result in pathological cardiac hypertrophy, characterized by increased cardiomyocyte size, cell death, fibrosis and reactivation of fetal gene expression.^1^ Cardiac hypertrophy leads to heart failure, which is a major health problem and accounts for inordinate mortality worldwide.^2^, ^3^ However, current therapies available for patients with cardiac remodelling, such as ACE inhibitors and β‐blockers,^4^, ^5^ do not fully meet the clinical needs. Therefore, the identification of novel protective agents is of great interest for improving preventive and therapeutic strategies.

AMP‐activated protein kinase (AMPK) is a kinase that plays a crucial role in cell growth regulation and mitochondrial function during metabolic stress.^6^ Accumulating evidence has suggested AMPK as an inhibitor of cardiac hypertrophy due to its inhibition on protein synthesis and reactive oxygen species (ROS) production.^7^, ^8^, ^9^, ^10^ Under physiological conditions, intracellular ROS are produced and cleared in an equilibrium state. When cardiomyocytes are exposed to pathogenic stimuli, ROS accumulate and stimulate intracellular signalling proteins with a critical role, such as mitogen‐activated protein kinase (MAPK). MAPK transduces signals to transcription factors and reactivates fetal gene expression.^11^, ^12^, ^13^

Thymoquinone (TQ, Figure S1) is a bioactive natural product mainly derived from *Nigella sativa* seeds (black cumin). It has been widely used in ethnomedicine to treat disorders including diabetes, cancer, rheumatism and neurological diseases.^14^ With regard to the cardiovascular system, several population and rodent studies have shown that TQ provides protection against doxorubicin‐induced cardiotoxicity,^15^ allows for substantial recovery of cardiac function after ischemia/reperfusion injury,^16^ and exhibits a blood pressure‐lowering effect.^17^ The primary mechanism underlying these effects may be its antioxidant activity, mediated by its ROS scavenger property and the preservation of endogenous antioxidants.^18^, ^19^

Although previous studies have explored the effect of *Nigella sativa* (the origin plant of which TQ is derived) on physiological cardiac hypertrophy,^20^, ^21^ it has not been yet elucidated whether this plant or TQ has a therapeutic effect on pathological cardiac hypertrophy. In this study, we tested the hypothesis that TQ may be a promising biological therapy to slow the progression of pathological cardiac hypertrophy. We also investigated the potential underlying mechanisms using a selective inhibitor of AMPK in in vivo and in vitro experiments.

## 2 MATERIALS AND METHODS

### 2.1 Antibodies and reagents

Antibodies against the following proteins were purchased from Cell Signaling Technology (USA): phosphorylated (p‐) AMPKα (Thr172) (used in experiments at 1:1,000 dilution), total AMPKα (at 1:1,000 dilution), p‐ACC (Ser79) (at 1:1,000 dilution), total ACC (at 1:1,000 dilution), p‐ERK1/2 (Thr202/Tyr204) (at 1:2,000 dilution), total ERK1/2 (at 1:2,000 dilution), p‐p38 (Thr180/Tyr182) (at 1:1,000 dilution), total p38 (at 1:1,000 dilution), p‐JNK1/2 (Thr183/Tyr185) (at 1:1,000 dilution), total JNK1/2(at 1:1,000 dilution), p‐mTOR (Ser2448) (at 1:1,000 dilution), total mTOR (at 1:1,000 dilution), p‐p70S6K (Thr389) (at 1:1,000 dilution), total p70S6K (at 1:1,000 dilution) and HRP‐linked secondary antibody (at 1:3,000 dilution). The antibody against GAPDH (at 1:3,000 dilution) was purchased from Abclonal. In addition, the following reagents were used: thymoquinone (TQ; Sigma‐Aldrich), phenylephrine (PE; Tokyo Chemical Industry) and compound C (CpC; Selleck).

### 2.2 Animals and treatment

All experimental animal procedures were approved by the Tab of Animal Experimental Ethical Inspection of the First Affiliated Hospital, Zhejiang University School of Medicine, Hangzhou, China, and were carried out in strict accordance with the Guidelines for the Care and Use of Laboratory Animals of the National Institutes of Health. The experimental animals were male C57BL/6J mice aged 8–10 weeks and weighing 22–24 g. They were purchased from the Experimental Animal Center of the First Affiliated Hospital and housed in this animal centre.

First, the mice were randomly assigned to one of the following four groups after 1 week of adaptation: sham operation + corn oil (sham + vehicle), sham operation + TQ (sham + TQ), transverse aortic constriction (TAC) operation + corn oil (TAC + vehicle) and TAC operation + TQ treatment (TAC + TQ). The TAC‐induced pressure overload model was established according to that described in a previous study.^22^ Mice in the sham group underwent a similar procedure without constricting the aorta. Two days after the operation, TQ (50 mg/kg, dissolved in corn oil) or the same volumes of corn oil were orally administrated once daily for six consecutive weeks.

Then, experiments were performed to examine the mechanism of action of TQ. Animals were randomly assigned to one of the following four groups: sham + vehicle, TAC + vehicle, TAC + TQ and TAC + TQ + CpC. TQ was administered at the same daily dose as previously described. The AMPK inhibitor CpC was administered intraperitoneally at 20 mg/kg/day.

At 6 weeks, mice underwent echocardiography and were sacrificed for the other experiments.

### 2.3 Transthoracic echocardiography

Six weeks after TAC or sham operation, all mice were anaesthetized with 4% chloral hydrate (0.01 ml/g, intraperitoneal injection). Subsequently, transthoracic echocardiography was performed by a skilled blinded technologist using a GE Vivid E95 ultrasound system (General Electric Company) equipped with an 18‐MHz probe. Data related to left ventricular (LV) chamber dimensions and wall thickness were extracted from the M‐mode traces.

### 2.4 Histological analysis

The ratios of heart weight/body weight (HW/BW) and HW/tibia length (HW/TL) were calculated to assess the severity of LV hypertrophy. The hearts were fixed in 4% paraformaldehyde immediately after isolation. After 16 h, the hearts were embedded with paraffin, followed by transverse slicing into 4‐μm sections. The slices were stained with haematoxylin and eosin, and Masson's trichrome following standard methods.^23^, ^24^ Ventricular sections were stained with wheat germ agglutinin (WGA) labelled with fluorescein isothiocyanate (FITC) to examine the cross‐sectional area of cardiomyocytes. The cell area and myocardial fibrosis were measured using Image J software (NIH).

### 2.5 Neonatal rat cardiomyocyte culture and treatment

Primary Neonatal rat cardiomyocyte (NRCMs) were isolated from neonatal (1–3 day) Sprague Dawley rats using a Neonatal Heart Dissociation Kit (Miltenyi Biotechnology) according to the manufacturer's instructions. Subsequently, the cardiomyocytes were kept in DMEM culture media containing 10% fetal bovine serum for 24 h under normal conditions (at 37°C with 5% CO_2_). This was followed by a change to serum‐free DMEM for 18 h. The cells were then pre‐incubated with the AMPK inhibitor CpC (5 µM) for 1 h and simultaneously treated with phenylephrine (PE, 50 µM) and TQ (5 µM) for another 24 h before they were harvested.

### 2.6 RNA isolation and quantitative real‐time PCR

Trizol reagent (Takara) was used to extract total RNA from ventricular tissues and NRCMs. HiScript II Q RT SuperMix (Vazyme) was used to perform reverse transcription. To quantify mRNA levels of genes, such as ANP, BNP and collagen I, qRT‐PCR was conducted using ChamQ Universal SYBR qPCR Master Mix (Vazyme). The PCR primer sequences of the target gene are reported in Table S1. GAPDH was used as an internal control. The comparative $Ct(2^{‐\Delta \Delta C_{T}})$ method was employed for subsequent analysis.

### 2.7 Western blot analysis

Total protein was isolated from ventricular tissues and NRCMs in lysis buffer (Beyotime Biotechnology). Equal amounts of protein (20–30 µg) were separated on 10% SDS/PAGE by electrophoresis. The proteins were subsequently transferred to polyvinylidene difluoride membranes (Millipore) and incubated with primary antibodies overnight at 4°C and secondary antibodies for 1 h at room temperature. All bands on blots were detected by chemiluminescence and analysed with ImageJ software.

### 2.8 Determination of oxidative stress

Dihydroethidium (DHE) staining was used to detect ROS in cardiac tissues as previously described.^25^ In brief, the ventricular tissues were frozen in liquid nitrogen immediately after isolation, and sliced into 5‐μm‐thick frozen sections. The slices were then stained with DHE and placed in a humidified room for 15 min at 37°C in the dark. A DCFH‐DA fluorescent probe (Beyotime Biotechnology) was used to measure ROS generation in NRCMs. The probe was diluted to 5 μM in serum‐free medium before use. After treatment, the cardiomyocytes were incubated with the probe for 20 min at 37°C in a CO_2_ incubator. Images of DHE staining and intracellular DCF fluorescence were captured by fluorescence microscopy (Olympus IX83). ImageJ software was employed to quantify the fluorescence intensity.

### 2.9 Measurement of cell surface area

NRCMs were stained with FITC‐Phalloidin (Yeasen Biotechnology) for myocyte size detection according to the manufacturer's protocol. To identify nuclei, fixed cells were counterstained with DAPI (Dawen Biotechnology). Immunofluorescent images were captured by an LSM 710 confocal microscope (Zeiss). Cell area was measured using ImageJ software.

### 2.10 Statistical analysis

Statistical analyses were conducted using the GraphPad Prism program (GraphPad). When data were normally distributed, one‐way ANOVA followed by Bonferroni post hoc test was performed to compare data from more than two groups. All data are presented as the mean ±SEM, and *p* < 0.05 was considered statistically significant.

## 3 RESULTS

### 3.1 TQ attenuated pressure overload‐induced cardiac failure and hypertrophy

**FIGURE 1:**
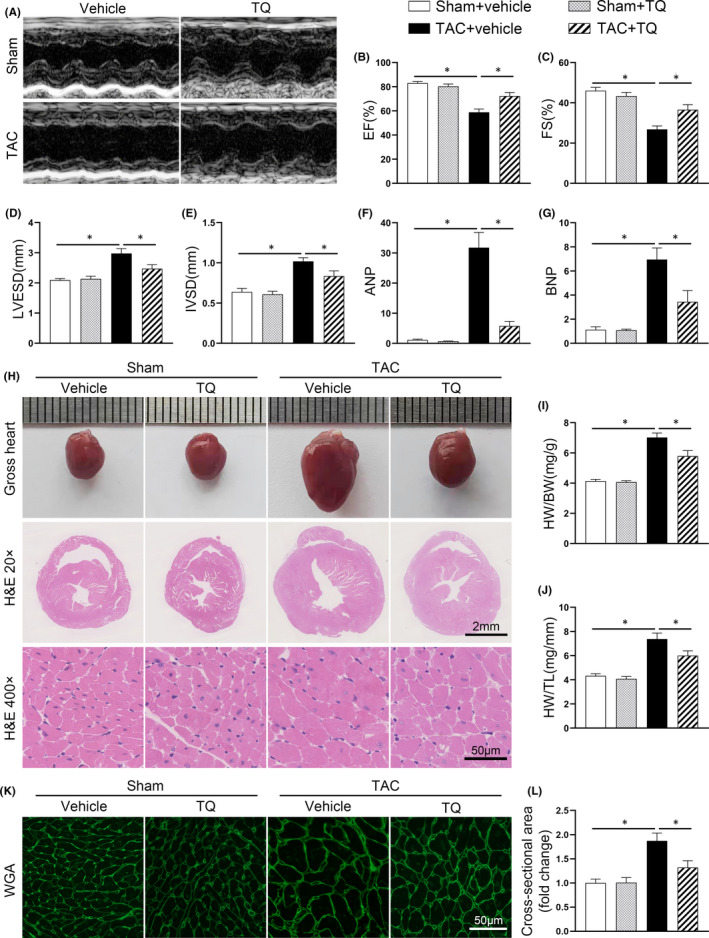
Thymoquinone (TQ) alleviated cardiac hypertrophy and dysfunction induced by TAC in vivo. (A) Representative M‐mode echocardiography of each group. (B–E) Measurement of echocardiographic parameters (*n* = 8). EF, ejection fraction; FS, fractional shortening; LVESD, left ventricular end‐systolic diameter; IVSD, diastole interventricular septal thickness. (F–G) Relative mRNA levels of ANP and BNP (*n* = 6). (H) Representative images showing gross cardiac morphology, transverse sections stained with haematoxylin and eosin and microscopic cross‐sections stained with haematoxylin and eosin. (I–J) Bar graphs showing quantitative data for heart weight (HW)/ body weight (BW) and HW/ tibial length (TL). (K) Histological examination of cardiac hypertrophy by FITC‐WGA staining. (L) Quantitative assessment of myocyte cross‐sectional area (*n* = 6). *p < 0.05; One‐way ANOVA followed by Bonferroni post hoc tests

TAC surgery was performed to establish a mouse model of cardiac dysfunction and hypertrophy. Based on a previous study,^26^ TQ (50 mg/kg/day) was administered by stomach gavage. Six weeks after TAC, echocardiography showed cardiac dysfunction in these mice as reflected by decreased LV ejection fraction (EF), LV fractional shortening (FS) and increased left ventricular end‐systolic diameter (LVESD) compared with sham‐operated mice (Figure 1A–D). TAC‐challenged mice also exhibited marked cardiac hypertrophy as illustrated by increased diastole interventricular septal thickness (IVSD) (Figure 1E). Although sham‐operated mice subjected to TQ administration had no echocardiographic changes, TAC‐operated mice treated with TQ exhibited preserved heart function and ameliorated cardiac hypertrophy (Figure 1A–E). All data of echocardiography are provided in Table S2. Next, we performed qRT‐PCR analysis to measure the mRNA levels of heart failure‐related biomarkers. There was a significant increase in atrial natriuretic peptide (ANP) and brain natriuretic peptide (BNP) mRNA levels after TAC. The abnormal mRNA expression of both markers is partially counteracted by TQ administration (Figure 1F–G), which is in line with the echocardiographic findings.

Histological analysis was performed to investigate the antihypertrophic effect of TQ. Treatment with TQ in TAC‐challenged mice showed significant improvement in gross morphology and observed in haematoxylin and eosin staining (Figure 1H) with concomitant reduction in the HW/BW and HW/TL ratios (Figure 1I–J). Next, we measured the average cross‐sectional area of cardiomyocytes by WGA staining. The transverse section of myocytes increased remarkably in comparison with the sham group 6 weeks after TAC surgery, while TQ administration resulted in normalization of this parameter (Figure 1K–L). These findings were in agreement with the histological observations.

### 3.2 TQ ameliorated cardiac fibrosis following TAC surgery

**FIGURE 2:**
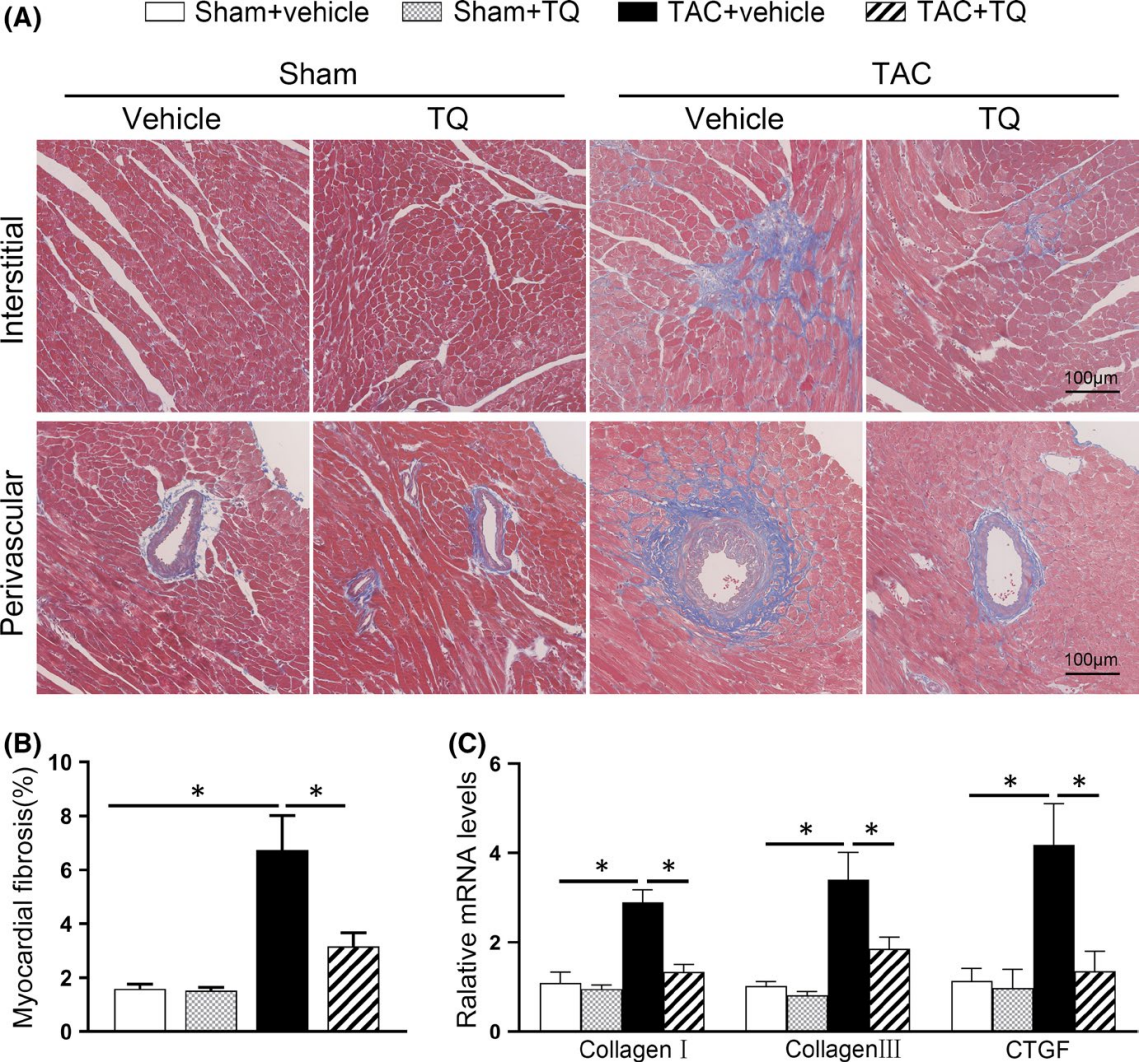
Administration of Thymoquinone (TQ) attenuated cardiac fibrosis induced by TAC in vivo. (A) Representative images of perivascular and interstitial myocardial fibrosis in the ventricular sections. (B) Bar graph showing quantified fibrotic areas (%) in Masson staining (*n* = 6). (C) Relative mRNA levels of collagen Ⅰ, collagen Ⅲ and CTGF (*n* = 6). *p < 0.05; One‐way ANOVA followed by Bonferroni post hoc tests

It is well‐documented that cardiac fibrosis typically occurs together with pathological hypertrophy and acts as an essential factor driving the progression of cardiac dysfunction.^27^ Here, myocardial interstitial and perivascular fibrosis in the ventricular tissues was evaluated in Masson's trichrome‐stained sections 6 weeks after the operation. TAC operation, as expected, promoted the progression of cardiac fibrosis, while treatment with TQ counteracted this alteration significantly (Figure 2A–B). In line with our previous results, the mRNA expression of collagen Ⅰ, collagen Ⅲ and CTGF were significantly increased in mice of the TAC group compared with their matched controls, but remarkably decreased in TQ‐treated mice in the TAC group (Figure 2C).

### 3.3 TQ inhibited TAC surgery‐induced oxidative stress.

**FIGURE 3:**
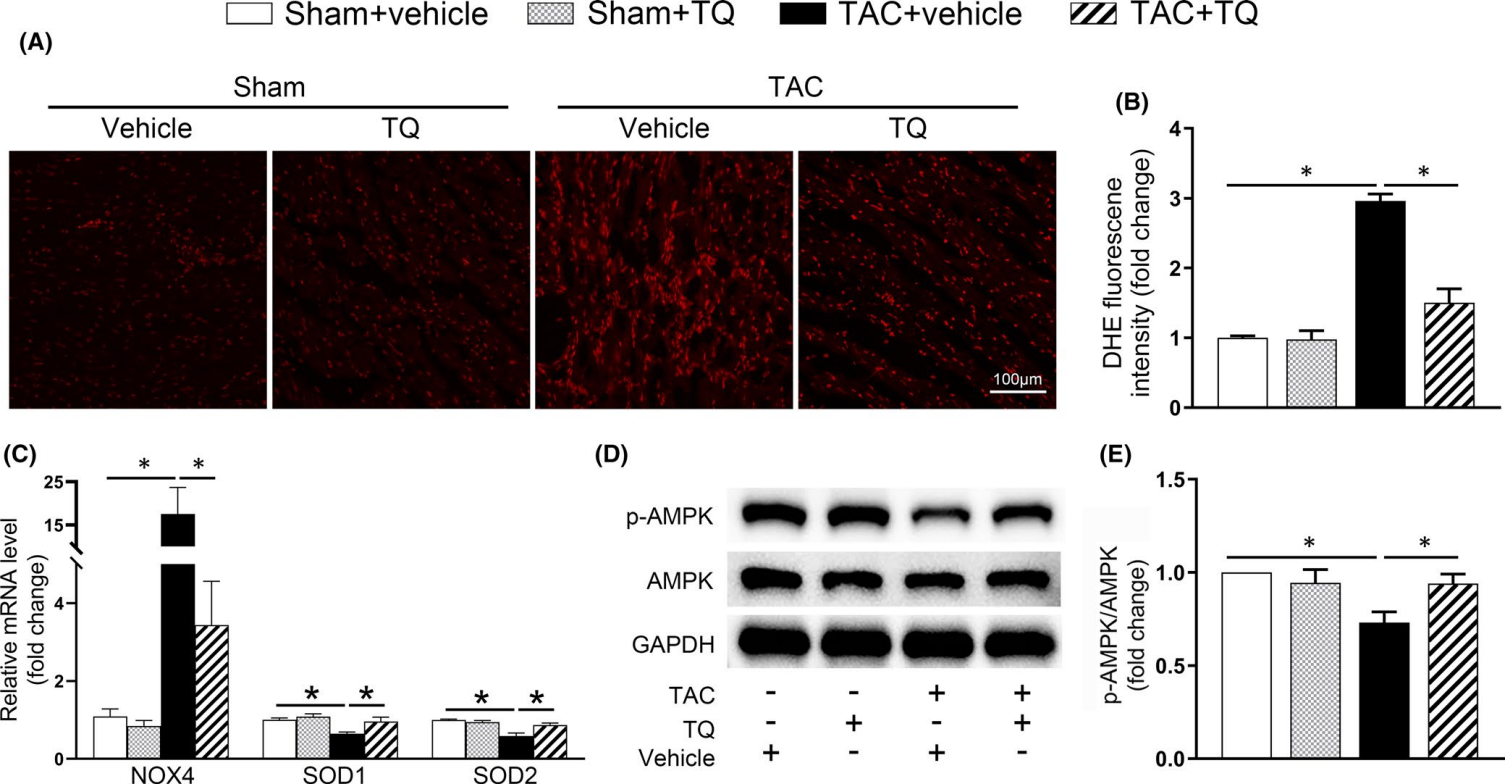
Administration of Thymoquinone (TQ) mitigated myocardium oxidative stress induced by TAC in vivo. (A–B) Representative images and quantification of DHE staining in ventricular sections (*n* = 6). (C) mRNA levels of NOX4, SOD1, SOD2 in the heart tissues of each group were examined by qRT‐PCR (*n* = 6). (D–E) Representative western immunoblots and quantitative analysis showing expression of phosphorylated (p‐) AMPK and total AMPK in mice heart tissues (*n* = 4). *p < 0.05; One‐way ANOVA followed by Bonferroni post hoc tests

Cardiac hypertrophy leads to increased intracellular ROS levels, which in turn exacerbate hypertrophy and fibrosis.^28^ Accordingly, we wondered whether TQ exhibits an anti‐oxidative stress effect in the development of myocardial hypertrophy. When examining DHE‐stained samples, we found that pressure overload for 6 weeks resulted in a substantial increase in ROS levels in ventricular tissues, while TQ treatment reduced levels close to those of the sham‐operated group (Figure 3A–B). In addition, the qRT‐PCR analysis revealed that TQ restored the mRNA expression of oxidant stress‐related genes (NOX4, SOD1 and SOD2) in mice of the TAC group (Figure 3C). Accumulating evidence has suggested that AMPK is an inhibitor of oxidative stress in cardiac hypertrophy.^9^, ^10^, ^29^ To confirm AMPK involvement in the mechanism of TQ, we next examined the phosphorylation level of AMPK using immunoblotting analysis. Our results showed that while TAC surgery remarkably inhibited AMPK phosphorylation, TQ administration restored AMPK phosphorylation to some extent in this pathological condition (Figure 3D–E).

### 3.4 TQ administration mitigated PE‐induced hypertrophic response in NRCMs

**FIGURE 4:**
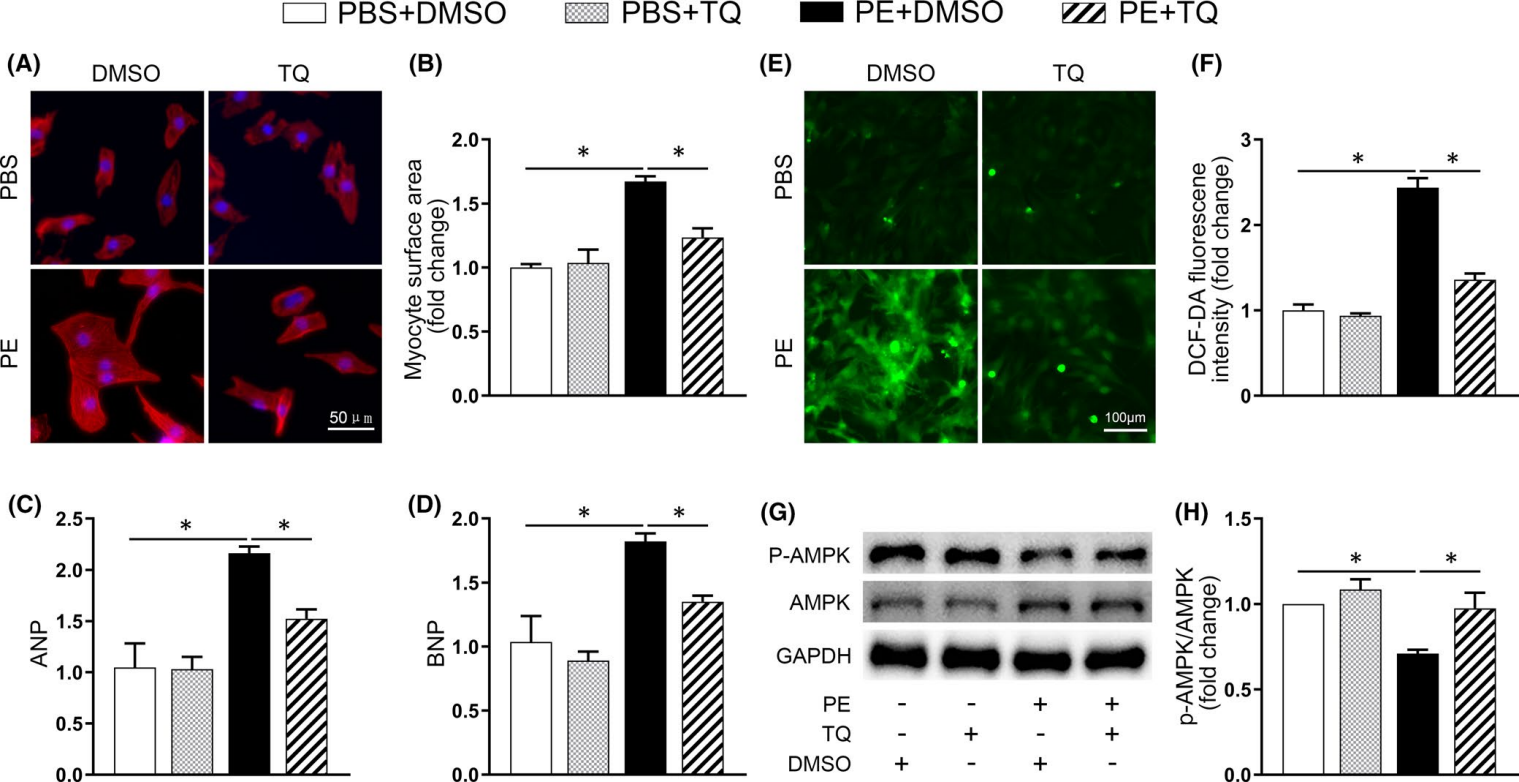
Thymoquinone (TQ) blocked the hypertrophic response in vitro. (A) Representative images of NRCMs used for the evaluation of cell surface area. Phalloidin/DAPI immunofluorescence staining was performed to identify cells. (B) Bar graph showing the normalized cell area (*n* = 3). (C–D) Relative mRNA expression of ANP and BNP in NRCMs of each group (*n* = 3). (E) Intracellular ROS in cardiomyocytes was detected using a DCFH‐DA fluorescent probe. (F) Quantitative analysis of DCFH‐DA fluorescence (*n* = 3). (G–H) Representative western immunoblots and quantitative analysis showing expression of phosphorylated (p‐) AMPK and total AMPK in NRCMs (*n* = 4). *p < 0.05; One‐way ANOVA followed by Bonferroni post hoc tests

We have demonstrated the efficacy of TQ in vivo, and next performed in vitro experiments using cardiomyocytes isolated from neonatal rats. First, a cell viability assay was performed to assess the effect of different concentrations of TQ on NRCMs (Figure S2). A concentration of 5 µM was chosen for subsequent in vitro experiments. The cells were simultaneously exposed to PE (50 µM) and TQ (5 µM) for 24 h. Total protein and RNA were isolated, and phalloidin staining as well as DCFH‐DA incubation (for ROS detection) were performed. We observed a protective effect of TQ on PE‐induced increased cell surface area (Figure 4A–B), and on expression of ANP and BNP (Figure 4C–D) and excessive ROS production (Figure 4E‐F). Similar to what was observed in vivo, TQ treatment resulted in restoration of PE‐mediated decrease of p‐AMPK (Figure 4G–H).

### 3.5 TQ lost its inhibitory effects on cardiac hypertrophy in the presence of CpC in vivo and in vitro

Given our observation that TQ enhanced the phosphorylation of AMPK under pathological conditions, we next used CpC, a specific AMPK inhibitor,^30^ to test the role of AMPK in mediating the beneficial effects of TQ on cardiac hypertrophy.

**FIGURE 5:**
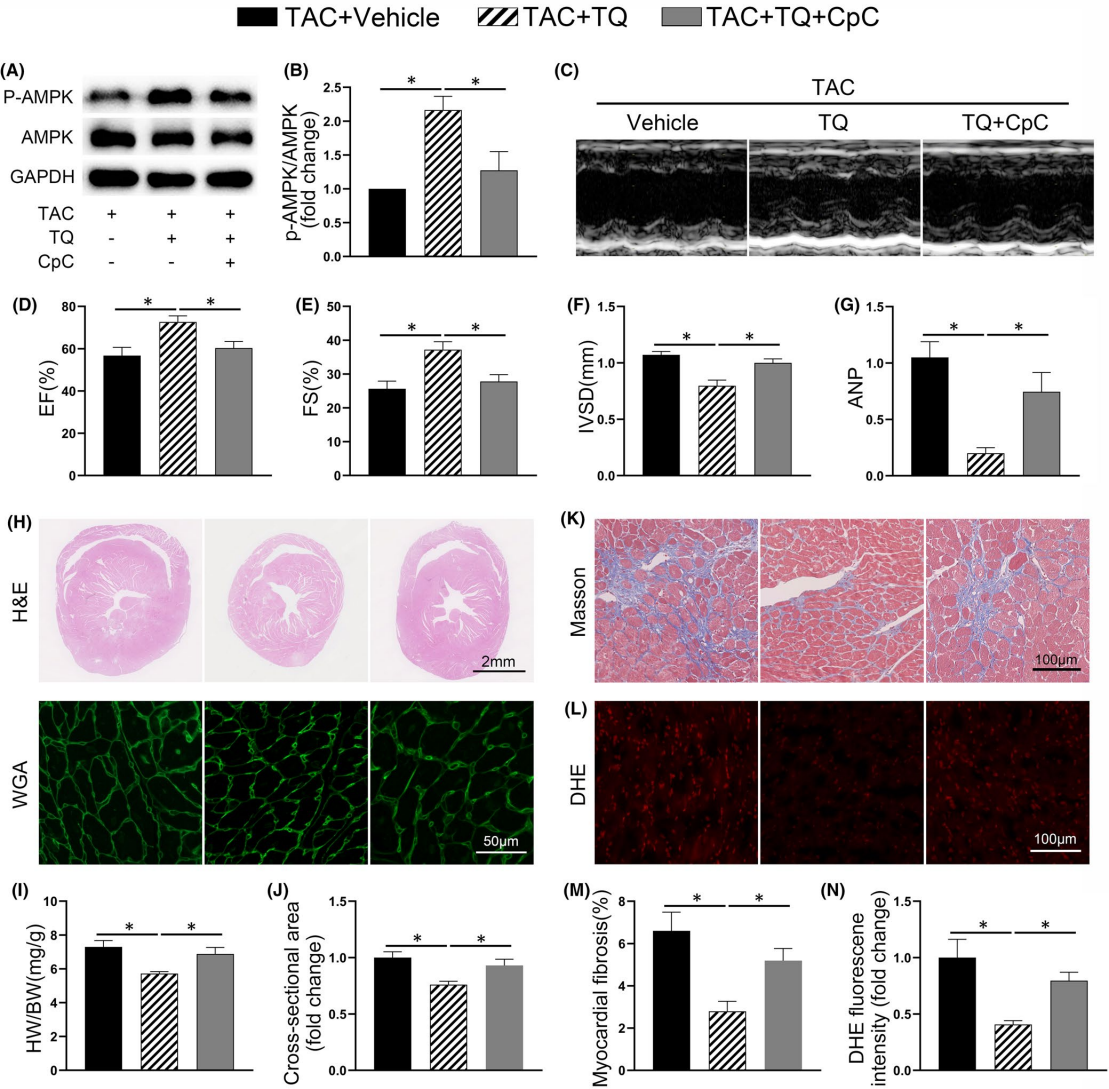
The effects of Thymoquinone (TQ) were partially counteracted by compound C (CpC) in vivo. (A–B) Representative western immunoblots and quantitative analysis showing expression of phosphorylated (p‐) AMPK and total AMPK in ventricular tissues (*n* = 4). (C) Representative M‐mode echocardiography of each group. (D–F) Measurement of echocardiographic parameters (*n* = 6). EF, ejection fraction; FS, fractional shortening; IVSD, diastole interventricular septal thickness. (G) Relative mRNA level of ANP (*n* = 6). (H) Representative images showing transverse sections stained with haematoxylin and eosin and FITC‐conjugated WGA. (I) Bar graphs showing quantitative data for heart weight (HW)/ body weight (BW) (*n* = 6). (J) Quantitative assessment of myocyte cross‐sectional area (*n* = 6). (K) Representative images of fibrosis in ventricular sections. (L) Representative images and quantification of DHE staining in ventricular sections. (M‐*N*) Bar graphs showing quantitative data for fibrosis and DHE staining respectively (*n* = 5–6). *p < 0.05; One‐way ANOVA followed by Bonferroni post hoc tests

As shown in Figure 5A–B, AMPK phosphorylation was no longer increased by TQ in TAC‐challenged mice after CpC injection. CpC significantly abolished the TQ‐mediated attenuation of TAC‐induced cardiac dysfunction and hypertrophy, as illustrated by echocardiographic measurements, fetal gene expression and morphological changes (Figure 5C–J). All data of echocardiography are provided in Table S3. In addition, the beneficial effects of TQ on cardiac fibrosis and oxidative stress were also partially abolished in mice treated with CpC (Figure 5K–N, Figure S3).

**FIGURE 6:**
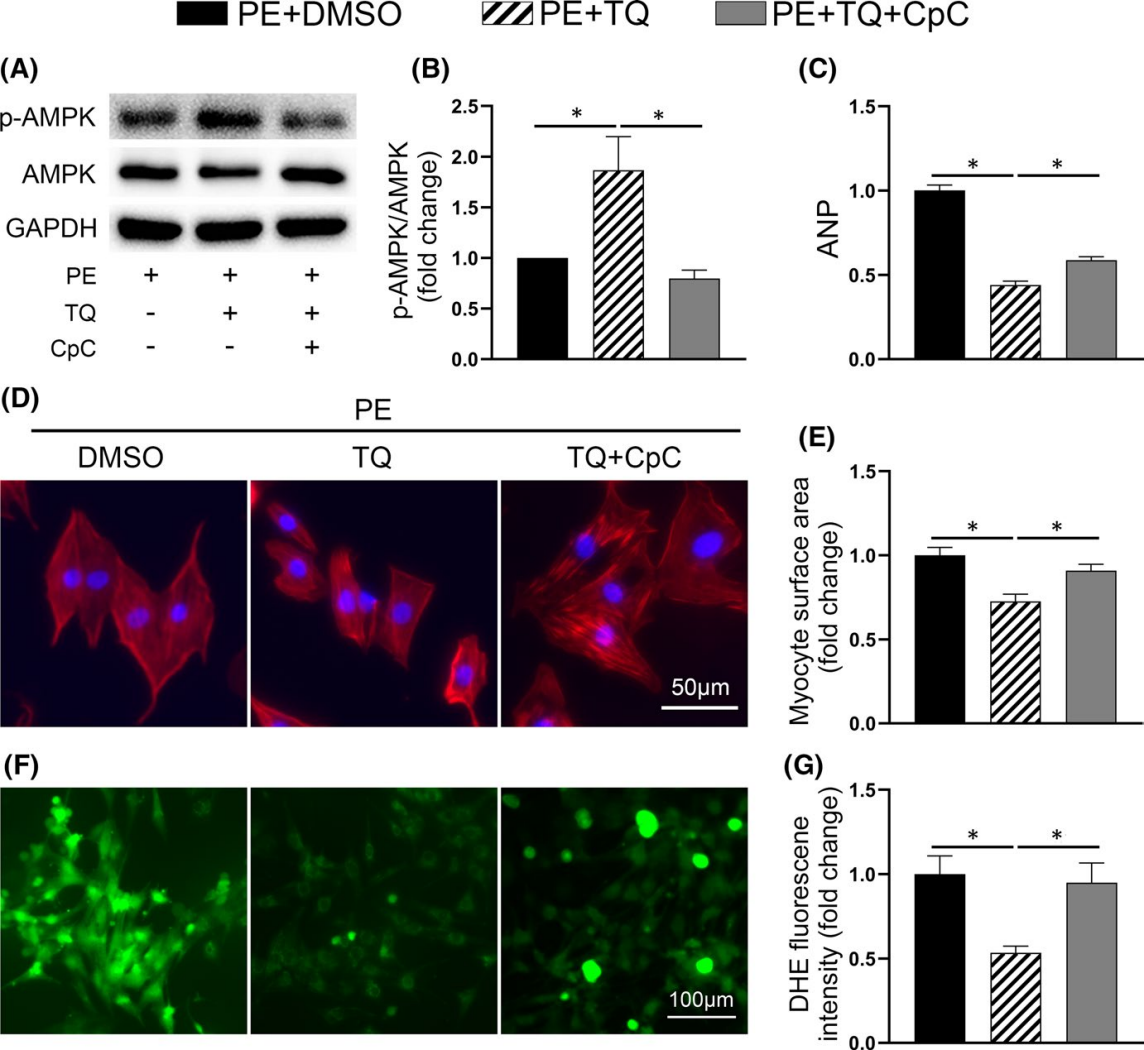
Compound C (CpC) abolished the antihypertrophic effects of Thymoquinone (TQ) in vitro. (A–B) Representative western immunoblots and quantitative analysis showing expression of phosphorylated (p‐) AMPK and total AMPK in NRCMs (*n* = 4). (C) Relative mRNA level of ANP (*n* = 3). (D) Representative images of NRCMs used for the evaluation of cell surface area. Phalloidin/DAPI immunofluorescence staining was performed to identify cells. (E) Bar graph showing the normalized cell area (*n* = 3). (F) Intracellular ROS in cardiomyocytes was detected using a DCFH‐DA fluorescent probe. (G) Quantitative analysis of DCFH‐DA fluorescence (*n* = 3). *p < 0.05; One‐way ANOVA followed by Bonferroni post hoc tests

Furthermore, TQ no longer enhanced the phosphorylation level of AMPK in NCRMs in the presence of CpC (Figure 6A–B). We observed that CpC neutralized the effect of TQ on inhibiting PE‐induced cardiomyocyte hypertrophy and fetal genes expression (Figure 6C–E). Importantly, the inhibitory effects of TQ on oxidative stress in NRCMs were greatly reduced when AMPK was inhibited (Figure 6F–G).

Altogether, these results support the hypothesis that TQ exerted its cardioprotective effect in an AMPK‐dependent manner.

### 3.6 TQ inhibited the MAPK signalling pathway in TAC‐ and PE‐induced cardiac hypertrophy, but CpC abolished this effect

To further elucidate the underlying molecular mechanisms of TQ, we evaluated signalling pathways playing a critical role in the hypertrophic process.

**FIGURE 7:**
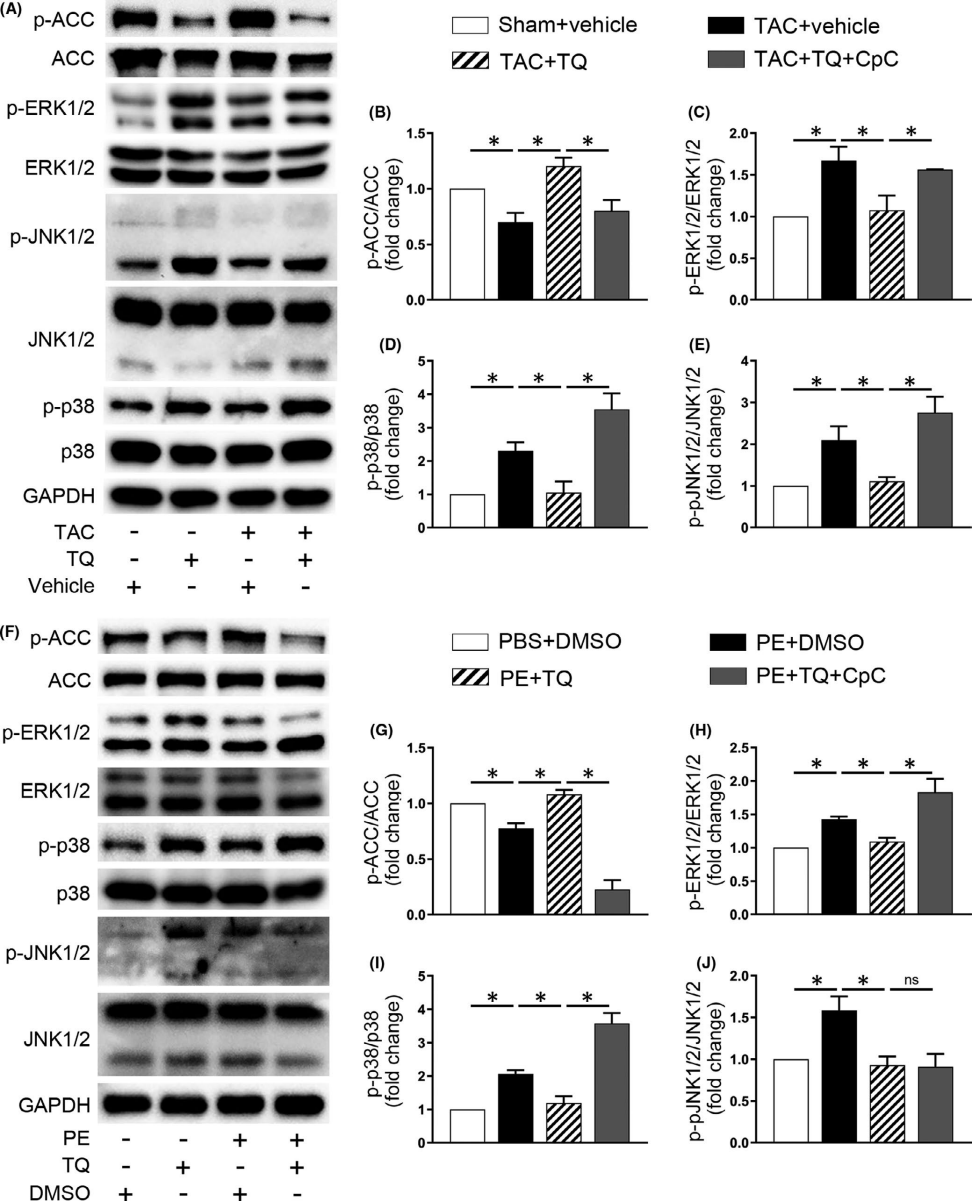
The effects of Thymoquinone (TQ) and compound C (CpC) on ACC and MAPK signalling pathway in vivo and in vitro. (A‐E) Representative western immunoblots(A) and quantitative analysis showing expression of phosphorylated (p‐) ACC(B), p‐ERK1/2(C), p‐p38(D) and p‐JNK1/2(E) in mice heart tissues (*n* = 4–6). (F–J) Representative western immunoblots (F) and quantitative analysis showing expression of p‐ACC(G), p‐ERK1/2(H), p‐p38(I) and p‐JNK1/2(J) in NRCMs (*n* = 3–4). *p < 0.05; ns, no significance; One‐way ANOVA followed by Bonferroni post hoc tests

First, we examined the phosphorylation level of ACC in mice hearts. ACC is one of the direct targets of AMPK and mirrors the activity of AMPK.^31^ Consistent with what was observed for p‐AMPK, phosphorylation of ACC was reduced in TAC‐injured hearts, and significantly restored in the TAC + TQ group, which further supported that TQ activated AMPK in the pressure‐overload model. CpC, as expected, remarkably downregulated phosphorylation of ACC (Figure 7A–B). Furthermore, 6 weeks after the TAC operation, ERK1/2, p38 and JNK1/2 MAPK were activated in the ventricular tissues compared with the sham group. These effects were, to some extent, counteracted by TQ. However, TQ‐mediated inhibition of the MAPK signalling pathway was abolished by CpC in vivo (Figure 7A, C–E). Similar results were observed when PE‐treated NRCMs were used (Figure 7F–J). Unexpectedly, inhibition of JNK1/2 phosphorylation following TQ treatment was not reversed by CpC in vitro (Figure 7J).

In addition, we investigated whether TQ affects the mammalian target of rapamycin (mTOR) and its effector p70S6K, members of a key downstream signalling pathway of AMPK in the progression of cardiac hypertrophy.^32^, ^33^ Here, we observed a marked increase in phosphorylation levels of mTOR and p70S6K in TAC‐injured heart and PE‐treated NRCMs. However, phosphorylation of these molecules was not significantly altered after TQ treatment (Figure S4).

In summary, these results suggested that AMPK/MAPK signalling plays a major role in the cardioprotective benefits of TQ treatment.

## 4 DISCUSSION

The present study demonstrates that TQ protects against cardiac remodelling and dysfunction in mice subjected to TAC. These observations were supported by our in vitro experiments in which the deleterious effect of PE was partially counteracted by TQ incubation in NRCMs. In other experiments, it was demonstrated that the AMPK/MAPK signalling pathway was involved in the protective mechanisms of TQ. Cardioprotective effects of TQ were markedly abolished by AMPK inhibition in vivo and in vitro.

A considerable amount of evidence provided support for the use of TQ in the cardiovascular system to treat atherosclerosis,^34^ hypertension,^35^ diabetic cardiomyopathy^26^ and dyslipidaemia.^36^ However, whether TQ prevents pathological cardiac hypertrophy remains elusive. In the present study, TAC operation was employed to mimic the disease process of cardiac hypertrophy. In this model, sustained excessive pressure load results in hypertrophy, cardiomyocyte apoptosis, oxidative stress and fibrosis.^10^, ^22^, ^37^ Here, we observed that the fetal gene expression program was re‐activated, cross‐sectional cell areas in ventricular tissues were increased and cardiac function was dramatically decreased 6 weeks after TAC. Importantly, our results indicated that TQ exhibited significant protective effects against TAC‐induced injury, as assessed by quantification of ANP and BNP mRNA expression, echocardiographic measurement and histological analysis. These findings suggested that TQ may be a promising therapy for cardiac hypertrophy and dysfunction.

Earlier studies have investigated the efficacy of *Nigella sativa* (NS; the origin plant of which TQ is derived) on physiological cardiac hypertrophy. Al‐Hariri, M. T. et al. demonstrated that 2 months of NS (800 mg/kg) oral supplementation in rats enhanced the cardiac contractile properties, while the cardiac workload or energy consumption remains unchanged.^20^ It was also found that supplementation of NS (800 mg/kg) to exercise training promoted global cardiac hypertrophy.^21^ In the present study, emphasis was placed on pathological hypertrophy. Our findings regarding enhanced cardiac function are consistent with the findings of the previous study, but the conclusions concerning myocardial hypertrophy differ from those of previous authors. We attributed this discrepancy to the different mechanisms of the two types of myocardial hypertrophy^1^ and because the two substances (NS and TQ) are not exactly the same compound.

Cardiac fibrosis is a hallmark of cardiac remodelling induced by sustained pressure overload. It is a late‐stage, irreversible process that increases heart wall stiffness, deteriorates contractile function and leads to chronic heart failure.^38^, ^39^ The anticardiac fibrotic effect of TQ has been suggested in previous studies in which animals were exposed to lipopolysaccharides (LPS) or lead.^40^, ^41^ Similarly, our observations demonstrated that TQ treatment slowed the progression of interstitial and perivascular fibrosis in mice hearts following TAC surgery.

Excessive ROS accumulation has been associated with a progression to cardiac fibrosis and end‐stage heart failure.^12^, ^13^, ^42^, ^43^ Targeting oxidative stress has been the subject of investigation in the preclinical setting and demonstrated highly promising results.^44^, ^45^, ^46^ As a bioactive natural compound, TQ is one of the agents that has been considered as potent antioxidants and has been extensively studied in the past decade. The current study demonstrated the protective effect of TQ on oxidative stress in vivo and in vitro, as shown by the detection of ROS‐related gene expression in addition to DHE/DCFH‐DA fluorescent microscopy. The free radical scavenger property of TQ could be ascribed to its quinone moiety and its capacity to pass cell membranes and enter subcellular organelles.^14^ The rebalance of the oxidative status following TQ treatment is presumed to translate into the recovery from pathological cardiac hypertrophy.

AMPK is a stress‐activated kinase that regulates cardiac metabolism, protein synthesis and the ROS/redox balance under physiological and pathological conditions.^8^, ^47^ There is ample evidence suggesting that AMPK is an inhibitor of pathological cardiac hypertrophy.^48^, ^49^, ^50^ On the other hand, previous studies have reported that AMPK activation can be regulated by TQ.^51^, ^52^ Therefore, we tested the hypothesis that AMPK plays a crucial role in mediating the cardioprotective benefits of TQ. Our results showed that TQ restored the phosphorylation of AMPK in our experimental models of cardiac hypertrophy, both in vivo and in vitro. However, when CpC (a selective AMPK inhibitor) was administered, TQ no longer protects the heart from hypertrophy, fibrosis and oxidative stress.

The MAPK family has been recognized as one of the downstream signal pathways of AMPK.^53^, ^54^ The family includes ERK1/2, p38 and JNK1/2, which all play a critical role in the progression of pathological cardiac hypertrophy.^55^ Earlier reports have shown that TQ inhibited MAPK signalling in models of cardiac damage and acute liver injury.^56^, ^57^ In line with these findings, we observed activation of ERK1/2, p38 and JNK1/2 MAPK in the post‐TAC cardiac tissues and PE‐treated NRCMs. TQ administration markedly inhibited these effects. However, TQ lost its inhibitory effects on MAPK signalling when AMPK was inhibited in vivo and in vitro, illustrating that TQ exerts its protective effect in an AMPK‐dependent manner. Unexpectedly, CpC did not enhance the phosphorylation level of JNK1/2 in vitro. We attributed this to the different conditions of pressure overload‐ and PE‐induced cardiac hypertrophy. This observation suggested that TQ may reduce JNK phosphorylation through other pathways in vitro. In addition, we examined phosphorylated mTOR and p70S6K, members of a key downstream signalling pathway of AMPK, in the progression of cardiac hypertrophy. However, we did not observe that TQ inhibited the phosphorylation of mTOR and p70S6K in vivo and in vitro. It is possible that the level of AMPK activation induced by TQ did not reach the threshold in our models. These observations are in line with results from a previous study, in which Gelinas, R. et al. found that a low level of AMPK activation prevented cardiomyocyte hypertrophy without inhibition of p70S6K phosphorylation.^48^

In conclusion, our study provides evidence of the protective effect of TQ on pathological cardiac hypertrophy through activation of AMPK and inhibition of MAPK signalling in vivo and in vitro (Figure S5). These results indicate the clinical relevance of TQ as a promising treatment strategy for hypertrophic diseases in the future.

## CONFLICT OF INTEREST

The authors confirm that there are no conflicts of interest.

## AUTHOR CONTRIBUTIONS

**Heng Chen:** Conceptualization (lead); Methodology (lead); Project administration (lead); Software (lead); Writing – original draft (lead). **Chengui Zhuo:** Methodology (equal); Project administration (equal); Software (equal). **Aohan Zu:** Methodology (equal); Project administration (equal). **Shuai Yuan:** Project administration (equal). **Han Zhang:** Visualization (equal). **Liangrong Zheng:** Conceptualization (supporting); Funding acquisition (lead); Writing – review & editing (lead). **Jianqiang Zhao:** Investigation (equal); Project administration (equal).

## Supporting information

Supplementary Material

## Data Availability

The data that support the findings of this study are available in the supplementary material of this article.
