# Supplementary material for: Thymoquinone ameliorates pressure overload‐induced cardiac hypertrophy by activating the AMPK signalling pathway
Source: J Cell Mol Med. 2021 Dec 24;26(3):855–67. doi: 10.1111/jcmm.17138 (PMC8817125; doi:10.1111/jcmm.17138)
Supplement: Supplementary file 1 — Supplementary Material [file JCMM-26-855-s001.docx]

**Thymoquinone** **ameliorates pressure overload-induced cardiac hypertrophy by activating the AMPK signaling pathway**

**Heng Chen^1^;** **Chengui Zhuo^2^; Aohan Zu^1^;** **Shuai Yuan^3^; Han Zhang^1^;** **Jianqiang Zhao^4^; Liangrong Zheng^1,*^**

**Supplementary Data**

**Supplementary Table S1.** Primers used for qRT-PCR analysis (5’-3’ orientation)

| Target | Species | Forward | Reverse |
| --- | --- | --- | --- |
| GAPDH | Mouse | AGGTCGGTGTGAACGGATTTG | GGGGTCGTTGATGGCAACA |
| ANP | Mouse | GTGCGGTGTCCAACACAGAT | TCCAATCCTGTCAATCCTACCC |
| BNP | Mouse | GAGGTCACTCCTATCCTCTGG | GCCATTTCCTCCGACTTTTCTC |
| Collagen Ⅰ | Mouse | GCTCCTCTTAGGGGCCACT | ATTGGGGACCCTTAGGCCAT |
| Collagen Ⅲ | Mouse | CTGTAACATGGAAACTGGGGAAA | CCATAGCTGAACTGAAAACCACC |
| CTGF | Mouse | GGCCTCTTCTGCGATTTCG | GCAGCTTGACCCTTCTCGG |
| NOX4 | Mouse | TGGCCAACGAAGGGGTTAAA | TCCTAGGCCCAACATCTGGT |
| SOD1 | Mouse | GGTCCACGAGAAACAAGATGA | CAATCACACCACAAGCCAAG |
| SOD2 | Mouse | CGGGGGCCATATCAATCACA | GCCTCCAGCAACTCTCCTTT |
| GAPDH | Rat | GGTGGACCTCATGGCCTACA | CTCTCTTGCTCTCAGTATCCTTGCT |
| ANP | Rat | GGGCTCCAATCCTGTCAATC | GCCGGTAGAAGATGAGGTCA |
| BNP | Rat | CTTGCGGAGGCGAGACAA | GCGCCAATCCGGTCTATCTT |

**Supplementary Table S2.** Echocardiographic parameters in mice after sham or TAC surgery.

| Parameter | Sham | | TAC | |
| --- | --- | --- | --- | --- |
|  | Vehicle | TQ | Vehicle | TQ |
| IVSD (mm) | 0.64±0.04 | 0.61±0.04 | 1.02±0.05^#^ | 0.84±0.07^#,^* |
| IVSS (mm) | 1.26±0.09 | 1.21±0.07 | 1.39±0.06 | 1.39±0.02 |
| LVPWD (mm) | 0.61±0.06 | 0.70±0.03 | 0.78±0.05 | 0.80±0.04# |
| LVPWS (mm) | 1.19±0.04 | 1.14±0.06 | 1.17±0.05 | 1.26±0.06 |
| LVEDD (mm) | 3.89±0.11 | 3.76±0.05 | 4.09±0.16 | 3.90±0.15 |
| LVESD (mm) | 2.09±0.05 | 2.13±0.09 | 2.98±0.16^#^ | 2.47±0.13* |
| EF (%) | 82.90±1.83 | 80.20±2.09 | 59.78±2.41^#^ | 72.25±3.00^#,^*  36.53±2.56^#,^* |
| FS (%) | 46.02±1.65 | 43.29±1.89 | 27.37±1.52^#^ |  |
| HR (min^-1^) | 481±13 | 464±15 | 506±17 | 494±31 |

Abbreviations: TQ, Thymoquinone; IVSD, diastole interventricular septal thickness; IVSS, systolic interventricular septal thickness; LVPWD, left ventricular posterior wall diastolic thickness; LVPWS, left ventricular posterior wall systolic thickness; LVEDD, left ventricular end‐diastolic diameter; LVESD, left ventricular end‐systolic diameter; EF, ejection fraction; FS, fractional shortening; HR, heart rate.

#, P < 0.05 compared to sham + vehicle group; *, P < 0.05 compared to TAC + vehicle group. n =8; One-way ANOVA followed by Bonferroni post-hoc tests.

**Supplementary Table S3.** Echocardiographic parameters in mice after TAC surgery.

| Parameter | TAC | | |
| --- | --- | --- | --- |
|  | Vehicle | TQ | TQ + CpC |
| IVSD (mm) | 1.07±0.03 | 0.80±0.05^#^ | 1.00±0.04* |
| IVSS (mm) | 1.54±0.05 | 1.52±0.10 | 1.41±0.10 |
| LVPWD (mm) | 0.88±0.07 | 0.88±0.07 | 0.84±0.05 |
| LVPWS (mm) | 1.21±0.05 | 1.31±0.05 | 1.25±0.10 |
| LVEDD (mm) | 4.07±0.22 | 3.92±0.23 | 4.11±0.13 |
| LVESD (mm) | 3.04±0.24 | 2.48±0.18 | 2.98±0.16 |
| EF (%) | 56.74±3.92 | 72.66±2.94^#^ | 60.31±3.15* |
| FS (%) | 25.63±2.31 | 37.19±2.38^#^ | 27.78±2.06* |
| HR (min^-1^) | 490±20 | 474±36 | 481±15 |

Abbreviations: TQ, Thymoquinone; CpC, compound C; IVSD, diastole interventricular septal thickness; IVSS, systolic interventricular septal thickness; LVPWD, left ventricular posterior wall diastolic thickness; LVPWS, left ventricular posterior wall systolic thickness; LVEDD, left ventricular end‐diastolic diameter; LVESD, left ventricular end‐systolic diameter; EF, ejection fraction; FS, fractional shortening; HR, heart rate.

#, P < 0.05 compared to TAC + vehicle group; *, P < 0.05 compared to TAC + TQ group. n =6; One-way ANOVA followed by Bonferroni post-hoc tests.

**Supplementary Figure S1.**


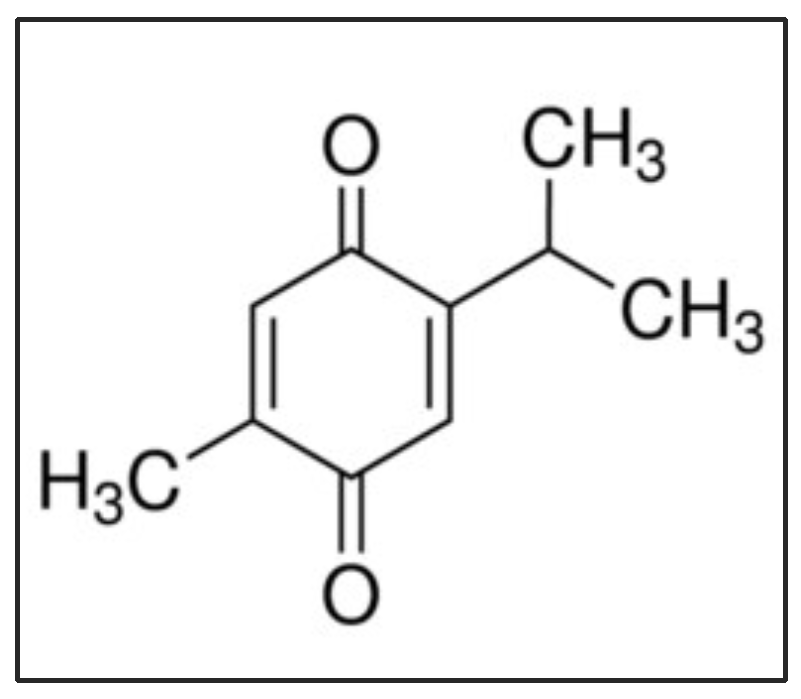


**Supplementary Figure S1.** Chemical structure of Thymoquinone (TQ).

**Supplementary Figure S2.**


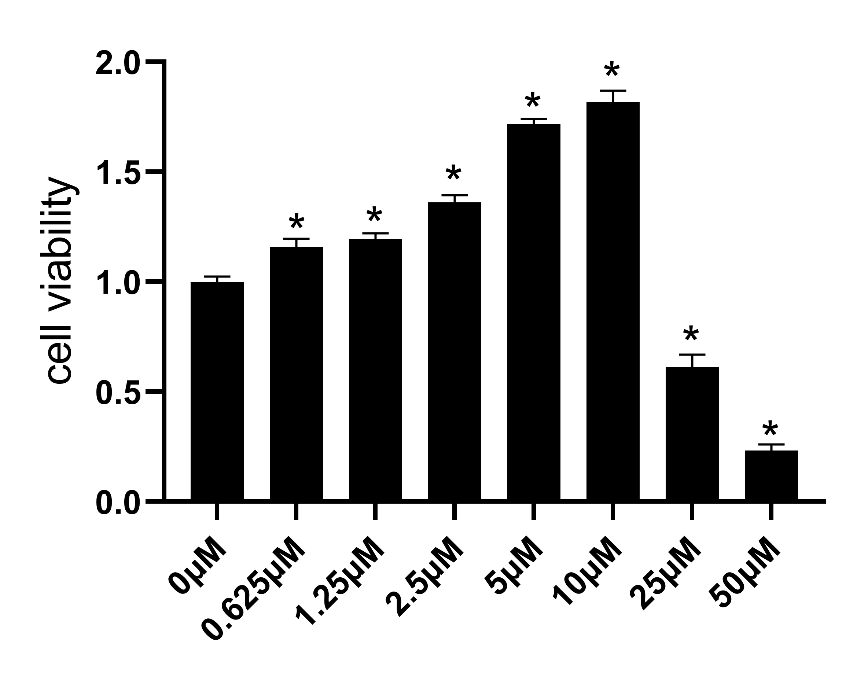


**Supplementary Figure S2.** Cell viability assay of different concentrations of TQ in Neonatal rat cardiomyocytes (NRCMs).

NRCMs were seeded onto a 96-well plate for 24h under normal condition (at 37 °C with 5% CO_2_), followed by different concentrations of TQ treatment for another 24 h. Afterward, cell viability was determined using a commercial Cell Counting Kit-8 (CCK-8; Biosharp, China) (n=5). We used 5 μM TQ to incubate with NRCMs in the following experiments. *p < 0.05 vs. the 0uM group; One-way ANOVA followed by Bonferroni post-hoc tests.

**Supplementary Figure S3.**


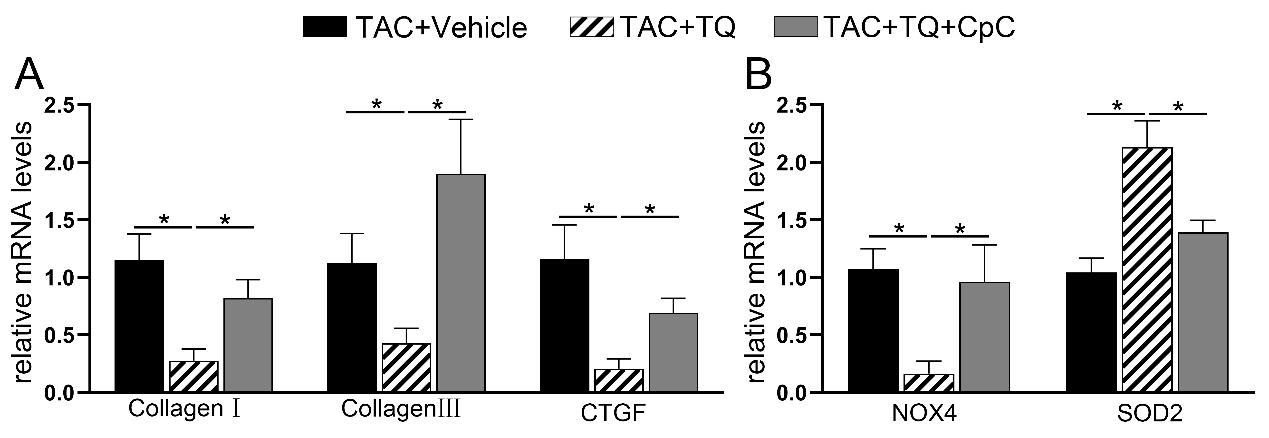


**Supplementary Figure S3.** The effects of Thymoquinone (TQ) and compound C (CpC) on mRNA expression of fibrosis-related genes(A) and oxidative stress-related genes(B) *in vivo*.

*, P < 0.05 compared to TAC + TQ group. n=6; One-way ANOVA followed by Bonferroni post-hoc tests.

**Supplementary Figure S4.**


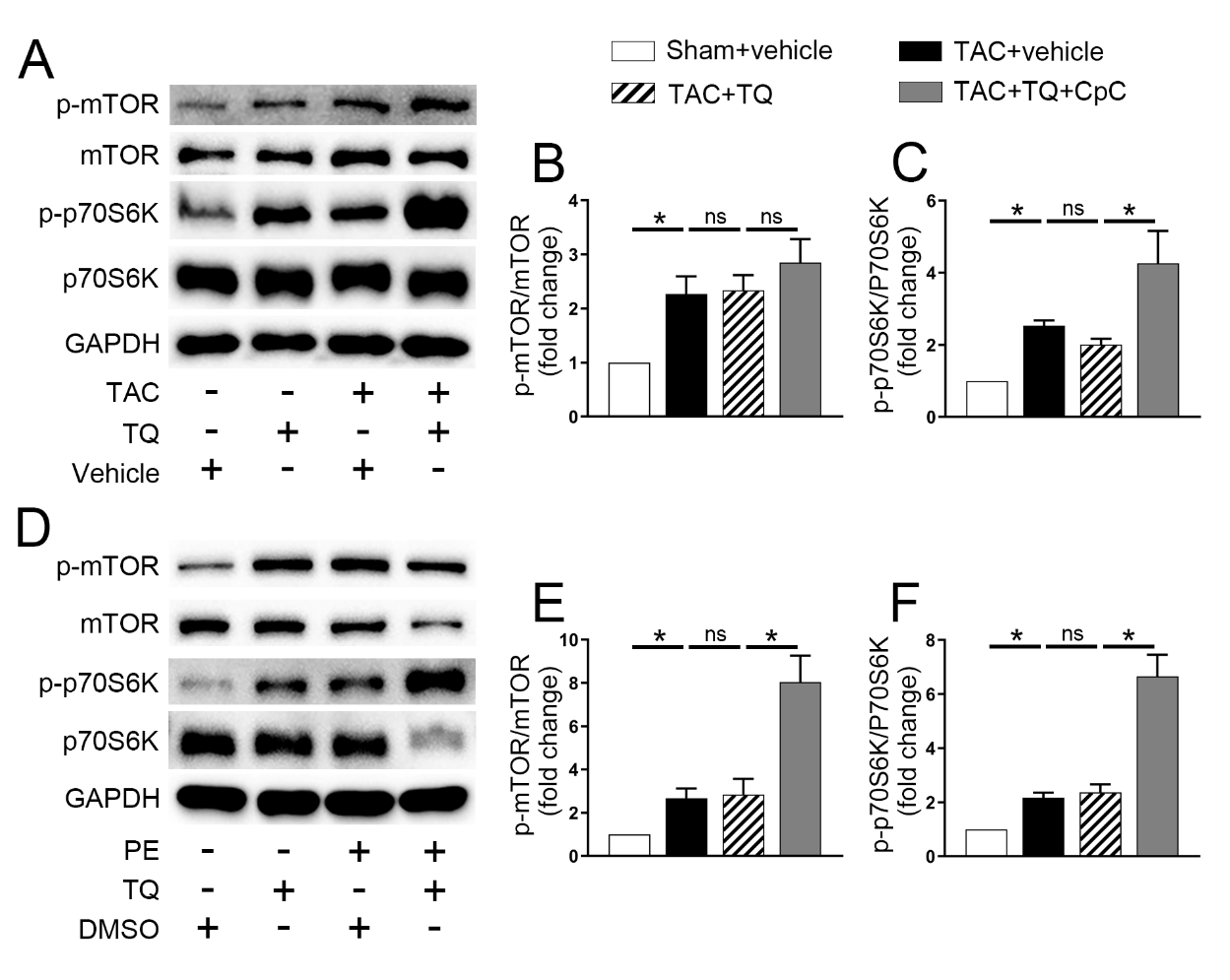


**Supplementary Figure S4.** The effects of Thymoquinone (TQ) and compound C (CpC) on mTOR signaling pathway *in vivo* and *in vitro*.

(A-C) Representative western immunoblots(A) and quantitative analysis showing expression of p-mTOR(B) and p-p70S6K(C) in mice heart tissues (n=4-6). (D-F) Representative western immunoblots(D) and quantitative analysis showing expression of p-mTOR(E) and p-p70S6K(F) in NRCMs (n=4). *P < 0.05; One-way ANOVA followed by Bonferroni post-hoc tests.

**Supplementary Figure S5.**


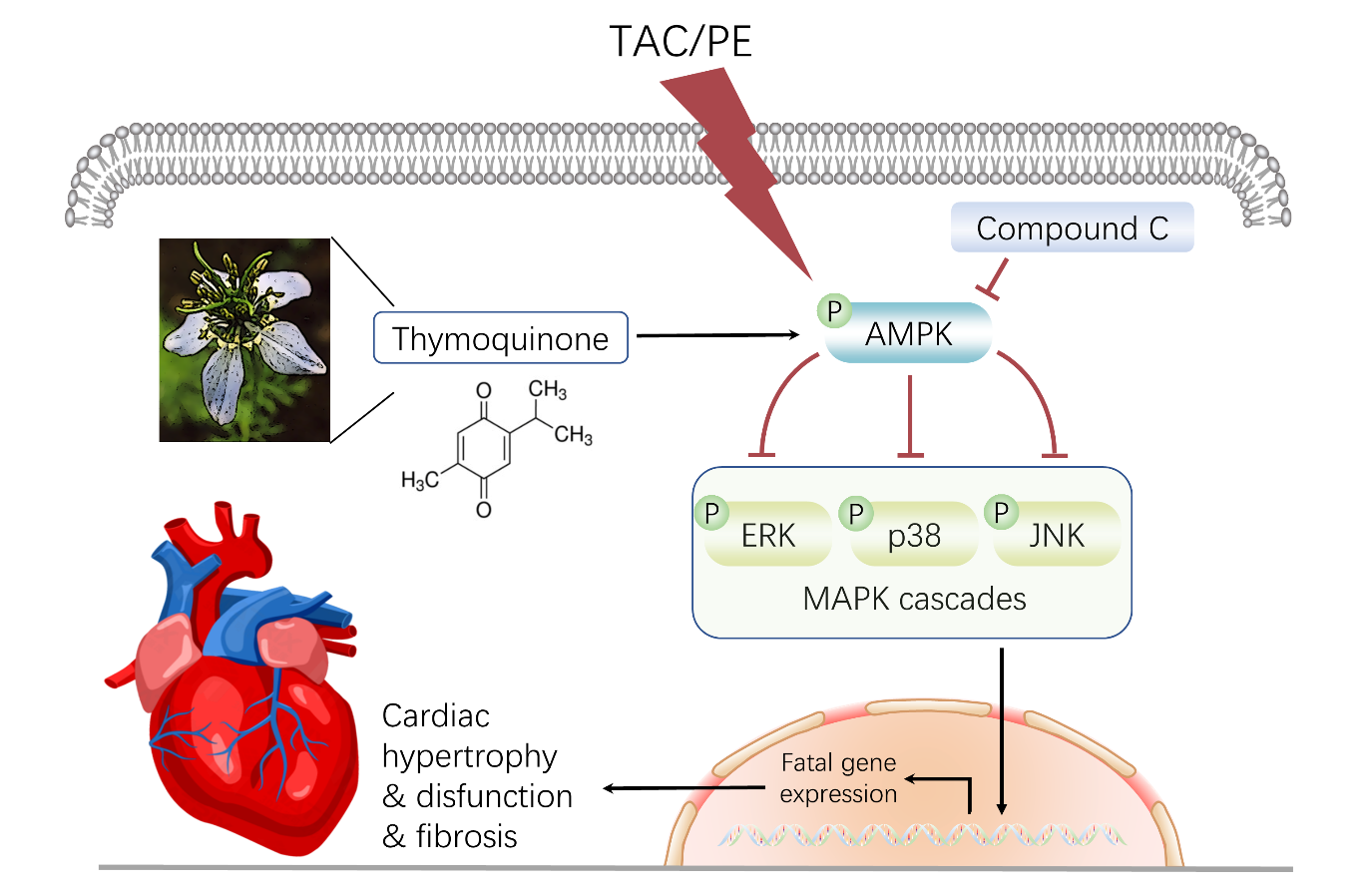


**Supplementary Figure S5.** Schematic diagram showing the molecular mechanisms underlying the TQ effects.
